# Fast skeletal troponin I, but not the slow isoform, is increased in patients under statin therapy: a pilot study

**DOI:** 10.11613/BM.2019.010703

**Published:** 2018-12-15

**Authors:** Alessandro Trentini, Savino Spadaro, Valentina Rosta, Maria C Manfrinato, Carlo Cervellati, Francesca Dalla Corte, Stefania Hanau, Carlo A Volta, Tiziana Bellini

**Affiliations:** 1 Section of Medical Biochemistry, Molecular Biology and Genetics, Department of Biomedical and Specialist Surgical Sciences, University of Ferrara, Ferrara, Italy; 2 Section of Anesthesia and Intensive Care, Department of Morphology, Surgery and Experimental Medicine, University of Ferrara, Ferrara, Italy

**Keywords:** statin, fast skeletal troponin, slow skeletal troponin, muscle damage, creatine kinase

## Abstract

**Introduction:**

Statin therapy is often associated with muscle complaints and increased serum creatine kinase (CK). However, although essential in determining muscle damage, this marker is not specific for skeletal muscle. Recent studies on animal models have shown that slow and fast isoforms of skeletal troponin I (ssTnI and fsTnI, respectively) can be useful markers of skeletal muscle injury. The aim of this study was to evaluate the utility of ssTnI and fsTnI as markers to monitor the statin-induced skeletal muscle damage.

**Materials and methods:**

A total of 51 patients (14 using and 37 not using statins) admitted to the intensive care unit of the University of Ferrara Academic Hospital were included in this observational study. Serum activities of CK, aldolase, alanine aminotransferase and myoglobin were determined by spectrophotometric assays or routine laboratory analysis. Isoforms ssTnI and fsTnI were determined by commercially available ELISAs. The creatine kinase MB isoform (CK-MB) and cardiac troponin I (cTnI) were evaluated as biomarkers of cardiac muscle damage by automatic analysers.

**Results:**

Among the non-specific markers, only CK was significantly higher in statin users (P = 0.027). Isoform fsTnI, but not ssTnI, was specifically increased in those patients using statins (P = 0.009) evidencing the major susceptibility of fast-twitch fibres towards statins. Sub-clinical increase in fsTnI, but not CK, was more frequent in statin users (P = 0.007). Cardiac markers were not significantly altered by statins confirming the selectivity of the effect on skeletal muscle.

**Conclusions:**

Serum fsTnI could be a good marker for monitoring statin-associated muscular damage outperforming traditional markers.

## Introduction

Hydroxymethylglutaryl-coenzyme A (HMG-CoA) reductase inhibitors, also known as statins, are one of the most prescribed cardiovascular disease (CVD) risk-reducing drugs worldwide, exerting this by the massive lowering of low density lipoprotein-cholesterol (LDL) concentration in blood (*1*). However, as every drug, statins come with adverse effects among which the most acknowledged is muscle toxicity, manifesting with mild muscle complaints such as muscle weakness, cramps, fatigue and only in rare severe cases, rhabdomyolysis (*2*). The frequency of such adverse effects is fairly low, ranging from 0.1% for severe events to 10-15% complaints of mild event; therefore, these drugs are perceived with a favourable safety profile considering that an impairment of muscular performance do not occur (*3*-*6*). A common clinical practice to reveal possible deleterious effects of statins on muscle is the increase in circulating creatine kinase (CK), where values greater than 1950 U/L (ten times the upper limit for normal values) are considered a reason for concerns (*7*).

Moreover, an accumulating body of evidence suggests that statin therapy increases CK activity even in the absence of muscle complaints (*4*, *6*). On the other hand, it has been established that muscle symptoms can also occur without CK elevations (*8*). Nonetheless, although CK is mainly represented in skeletal muscle, it cannot be considered as a specific marker since it is highly variable from subject to subject making it difficult to establish normal values, and it is prone to lifestyle-dependent changes (*9*, *10*). In addition to myopathy, cardiac or neurologic diseases can be possible causes for elevated CK found in serum, highlighting the volatile nature of this marker for muscle-related disorders (*10*, *11*). In addition to CK, other non-specific muscle markers exist spanning from aldolase and aspartate aminotransferase (AST) to myoglobin, which is mainly present in cardiac muscle and oxidative type I fibres (*12*, *13*). Although in the past they have been extensively used for diagnosis of myocardial infarction, they have been replaced by more specific cardiac markers such as the MB isoform of CK (CK-MB) and, particularly, cardiac troponin I (cTnI), which is a more sensitive and specific marker (*14*, *15*).

Thanks to base research, also more specific circulating markers for skeletal muscle emerged, making even possible to study the damage to different type of fibres. Indeed, skeletal troponins, mostly the slow-twitch and fast-twitch troponin I (ssTnI and fsTnI, respectively), have proven to be good candidate biomarkers for the evaluation of damage to slow oxidative (Type I) and fast glycolytic (Type II) fibres, respectively, following extensive exercise (*16*, *17*). In addition, the quantification of these isoforms by western blot or enzyme-linked immunosorbent assays (ELISA), alone or in combination, have been used to study the muscle damage within different pathological settings (*18*-*20*). Nonetheless, their application for studying and monitoring the statin-associated muscle damage is still scarce, with the only exception of animal studies (*21*).

Therefore, the purpose of the present work was to evaluate the utility of ssTnI and fsTnI as possible markers to monitor the statin-induced skeletal muscle damage. Furthermore, less specific markers such as aldolase, AST, myoglobin and CK, as well as cardiac markers like CK-MB and cTnI, were also evaluated.

## Materials and methods

### Patient selection

In this cross-sectional observational study, a total of 51 consecutive patients enrolled within the project “Diaphragmatic dysfunction in critically ill patients undergoing mechanical ventilation” and admitted to the intensive care unit (ICU) of the University of Ferrara Academic Hospital from 2014 to 2017 were included in the study. After patient recruitment two groups were formed: 14 patients under statin therapy and 37 patients without statin therapy. The study was approved by the local ethics committee, conforms to The Code of Ethics of the World Medical Association (Declaration of Helsinki) and was conducted according to the guidelines for Good Clinical Practice (European Medicines Agency). Written informed consent was obtained from each patient or next of kin prior to inclusion in the study. The inclusion criteria were: age 18 years or older, with expected mechanical ventilation for at least 72 hours. The exclusion criteria were history of neuromuscular disease, diaphragm atrophy or paralysis, abnormal values of cardiac markers, current thoracotomy, pneumothorax or pneumo-mediastinum, hypoxemia requiring FIO_2_ greater than 60%, presence of bronchopleural air leaks, pregnancy.

### Serum sampling

Serum samples were obtained from the collection of venous blood in anticoagulant-free tubes by centrifugation at 3000 rpm for 10 minutes after clotting, and stored aliquoted at - 80 °C until assay. In order to avoid possible loss of bioactivity, samples were analysed within 3 months from the collection and thawed only once.

### Quantification of skeletal troponins

Slow skeletal Troponin I (TNNI1 or ssTnI; Mybiosource, Cat. No. MBS2510383) and fast skeletal Troponin I (TNNI2 or fsTnI; Mybiosource, Cat. No. MBS927961) were assayed by commercially available ELISA kits according to manufacturer’s instructions. All reagents and standards were included in the kits and analysts were blinded for any clinical information. Briefly, undiluted serum samples were analysed in duplicate into 96-microwell microtiter plates precoated with anti-TNNI1 or anti-TNNI2 antibodies. Seven serial dilutions of TNNI1 standard (range of 46.8 - 3000 pg/mL) or TNNI2 standard (range 6.25 - 400 pg/mL) were dispensed on each plate in duplicate and incubated at 37 °C (90 minutes for TNNI1 and 2 hours for TNNI2). At the end of the incubation, the plates were emptied and 100 μL of biotinylated detection antibody working solution were added to each wells and incubated for 1 hour at 37 °C. After 3 washing cycles, 100 μL of streptavidin-horseradish peroxidase (HRP) conjugate working solution were added to each wells and the plates were incubated for further 30 minutes at 37 °C and then washed 5 times. Finally, 90 μL of substrate solution were added to each well and the plates incubated at 37 °C for 30 minutes. The reaction was stopped by the addition of 50 μL of Stop Solution and the absorbance read at 450 nm. Concentrations of TNNI1 or TNNI2 were determined by interpolation from the standard curve. Both intra-assay and inter-assay coefficient of variations (CV) were below 10%. The samples were assayed in four different runs together with three internal quality controls to assess the performance of the kit and to correct for possible run-to-run variability. According to the manufacturer’s booklet and to our independent experiments (see Supplementary Table 1), cross-reactivity between ssTnI and fsTnI and analogues (cTnI) was negligible (less than 0.1%) or not present.

### Myoglobin, CK, CK-MB and cardiac Troponin I determinations

Myoglobin (Beckman Coulter, Cat. No. OSR6168 and Cat. No. 973243), CK (CPK; CK-Nac, Beckman Coulter, Cat. No. OSR6179 and OSR6279), CK-MB (CK-MB, Beckman Coulter, Cat. No. OSR61155), cardiac Troponin I (cTnI; AccuTnI+3, Beckman Coulter, Cat. No. A98143) were determined by routine analysis on Beckman Coulter automatic analysers at the Laboratory analysis of Sant’Anna Hospital, Ferrara, Italy.

### Aldolase, AST, ALT assays

Aldolase, AST and alanine aminotransferase (ALT) activities were assayed in undiluted serum samples by coupled spectrophotometric enzymatic assays on a Tecan Infinite M200 (Tecan Group Ltd., Männedorf, Switzerland). All enzymatic tests were performed following IFCC (International Federation of Clinical Chemistry and Laboratory Medicine) procedures.

### Statistical analysis

The normality of distribution of dependent variables was checked by Shapiro-Wilk test. Since the variables were not normally distributed, they were transformed to logarithmic function in order to approximate a normal distribution. In this way, the residuals of the ANCOVA model used for further statistical analyses were normally distributed. To correct for possible confounding factors such as age, gender, and body mass index (BMI), two group comparisons were performed on log-transformed variables by ANCOVA and including the listed variables as covariates and the other biological variables (CK, ssTnI, fsTnI *etc.*) as outcomes. Comparisons not corrected for confounding factors were performed on non-transformed variables by the non-parametric Mann-Whitney U test. Fisher’s exact or Chi-square test were used to compare the general characteristics of the samples or the proportion of abnormal values expressed as categorical variables. Abnormal values were determined based on a 75% cut-off for the circulating markers of muscular functionality/damage in the whole study population: if proteins were increased (> 75% cut-off) the values were considered as “sub-clinically abnormal”. Spearman’s rank test was used to analyse bivariate correlations. All the statistical analyses were performed by SPSS 21 (IBM), and an alpha level of 0.05 was considered statistically significant. The figures were made with Graphpad Prism v5.

## Results

Table 1 provides a summary of the demographic and main clinical characteristics of the study population. Patients using statins and not using the LDL lowering drugs were not different in age, female gender prevalence and BMI. In addition, there were no differences in the type of admission between the two groups. On the contrary, patients using statins had higher prevalence of CVDs, including hypertension and metabolic diseases (Table 1). However, after excluding hypertension from the CVDs their prevalence was not significantly different between the two groups (see Table 1, P = 0.198).

**Table 1:** Clinical and demographic characteristics of the study population

	**Statin users**	
	**NO** **(N = 37)**	**YES** **(N = 14)**
Age, years	70 (58)	72 (25)
Female, proportion	13/37	5/14
BMI (kg/m^2^)	27.8 (24.9)	26.7 (10.5)
**Type of statin (proportion)**		
Atorvastatin	-	9/14
Simvastatin	-	3/14
Rosuvastatin	-	1/14
Fluvastatin	-	1/14
**Type of admission (proportion)**		
Medical	20/37	5/14
Surgical	15/37	8/14
Trauma	2/37	1/14
Comorbidities (proportion)*		
Pulmonary diseases	9/37	2/14
Cardiovascular diseases (including hypertension)	23/37	13/14^†^
Cardiovascular diseases (excluding hypertension)	11/37	7/14
Chronic renal failure	3/37	3/14
Neurological diseases	6/37	4/14
Metabolic diseases	9/37	10/14^‡^
Cancer	11/37	4/14
Continuous variables (age and BMI) are expressed as median (range); categorical variables as frequencies and proportions. BMI: Body Mass Index. *Some patients are characterized by the concurrent presence of several comorbidities (*e.g.* pulmonary plus cardiovascular diseases). ^†^Fisher’s exact test, P = 0.041. ^‡^Fisher’s exact test, P = 0.002. P < 0.05 was considered statistically significant.		

**Figure 1:**
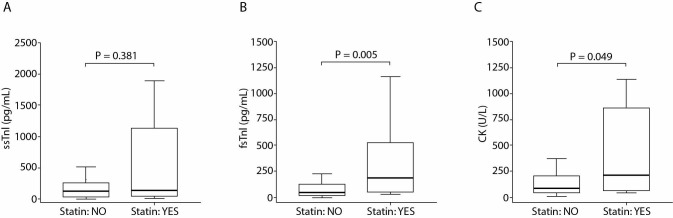
Median and interquartile range of the raw values of ssTnI, fsTnI and CK measured in the two groups. The concentration of ssTnI (panel A) was not different between the two groups whereas fsTnI (panel B) was significantly higher (P = 0.005) in subjects using statins. The same was observed for CK (panel C) (P = 0.049). The boundaries of the box represent the 25^th^-75^th^ quartile. The line within the box indicates the median. The whiskers above and below the box represent the highest and the lowest values excluding outliers. The P-values reported in the graph represent the exact value obtained by the Mann-Whitney U test without correcting for possible confounding factors.

Group comparisons were performed as stated in the Material and methods section. The raw data are reported in Supplementary Table 2. The crude and adjusted geometric means with the associated 95% confidence interval (95% CI) are reported in Table 2. Creatine kinase was significantly higher in statin compared to no statin using subjects (Table 2 and Figure 1), whereas aldolase and AST did not significantly differ between the two groups. Creatine kinase remained significantly higher in subjects using statins upon correction for covariates (*i.e.* age, sex, BMI) (Table 2). Furthermore, fsTnI, but not ssTnI, was higher in patients using statins (Table 2; Figure 1A and 1B), with values exceeding more than five times those measured in the NO statin cohort. Of note, upon adjustment this difference remained statistically significant (Table 2, adjusted means).

**Table 2:** Crude and adjusted means of the biochemical parameters determined in the study population

	**Statin use, crude means**			**Statin use, adjusted means**		
	**NO** **(N = 37)**	**YES** **(N = 14)**	**P**	**NO** **(N = 37)**	**YES** **(N = 14)**	**P**
**CK (U/L)**	88.9 (55.8 - 141.9)	241.0 (114.6 - 507.0)	0.027	89.7 (54.7 - 147.2)	247.7 (111.7 - 548.3)	0.024
**Aldolase (U/L)**	4.6 (3.8 - 5.6)	5.5 (4.0 - 7.5)	0.343	4.4 (3.6 - 5.3)	5.5 (4.0 - 7.5)	0.224
**AST (U/L)**	1.7 (0.9 - 2.9)	3.1 (1.3 - 7.5)	0.248	1.4 (0.8 - 2.6)	3.2 (1.3 - 8.1)	0.134
**ssTnI (pg/mL)**	82.0 (46.7 - 144.2)	163.3 (62.9 - 422.7)	0.217	79.4 (43.0 - 146.5)	180.3 (64.4 - 503.5)	0.161
**fsTnI (pg/mL)**	33.3 (17.1 - 65.2)	182.4 (62.5 - 533.3)	0.009	41.0 (21.4 - 78.3)	158.8 (55.8 - 452.9)	0.029
**Myoglobin (ng/mL)**	111.4 (64.7 - 191.9)	400.0 (167.5 - 959.4)	0.016	121.3 (67.9 - 216.8)	340.4 (132.7 - 872.9)	0.068
**cTnI (ng/mL)**	0.06 (0.03 - 0.12)	0.21 (0.08 - 0.51)	0.032	0.07 (0.04 - 0.13)	0.15 (0.06 - 0.37)	0.144
**CK-MB (ng/mL)**	4.6 (2.9 - 7.3)	10.2 (4.8 - 21.7)	0.077	4.58 (2.8 - 7.6)	11.2 (4.9 - 25.4)	0.074
Data represented as geometric crude and adjusted means with corresponding 95% confidence interval (95% CI) of the biochemical parameters obtained from the ANCOVA including sex, age and BMI as covariates. CK - creatine kinase. AST - aspartate aminotransferase. ssTnI - slow skeletal troponin I. fsTnI - fast skeletal troponin I. cTnI - cardiac troponin I. CK-MB - Creatine kinase-MB isoform. Covariates appearing in the adjusted model are evaluated at the following values: age = 70; sex = 0.33; BMI = 27.8 kg/m^2^. P < 0.05 was considered statistically significant.						

Finally, among the cardiac damage markers, only myoglobin and cTnI were found to be significantly higher in patients using statins (Table 2, crude means), differences that disappeared after correction for confounding factors (*i.e* age, sex, BMI).

Abnormal values were calculated as stated in the Material and methods section and the results are reported in Table 3. As shown, the frequency of sub-clinically abnormal serum values of all the examined markers was not different between the two groups with the exception for fsTnI. Indeed, a large part of patients using statins had sub-clinically abnormal values of circulating fsTnI compared to a lower proportion in those not using the lipid lowering drugs (Table 3).

**Table 3:** Percentage of subjects using or not using statin with sub-clinically abnormal (higher than 75% cut-off) serum muscle functionality/damage markers

	**Statin use**		
	**NO (%)**	**YES (%)**	**P**
**CK**	18.2	38.5	0.147
**Aldolase**	21.6	28.6	0.602
**AST**	21.6	28.6	0.602
**ssTnI**	21.6	28.5	0.506
**fsTnI**	13.9	50.0	0.007
**Myoglobin**	16.1	41.7	0.075
**cTnI**	16.7	41.7	0.086
**CK-MB**	19.4	33.3	0.330
CK - creatine kinase. AST - aspartate aminotransferase. ssTnI - slow skeletal troponin I. fsTnI - fast skeletal troponin I. cTnI - cardiac troponin I. CK-MB - Creatine kinase-MB isoform. The cut-off values used for the determination of the frequency of abnormal values were as follows: ssTnI, 320 pg/mL; fsTnI, 187 pg/mL; Myoglobin, 293 ng/mL; cTnI, 0.192 ng/mL; CK-MB, 9.3 ng/mL; CK, 276 U/L; Aldolase, 6.1 U/L; AST, 5.6 U/L. P < 0.05 was considered statistically significant.			

We first correlated the biochemical parameters with clinical and demographical characteristics of patients measured in the whole population and the results are presented in Table 4. None of the examined parameters correlated with BMI and only cTnI was significantly and positively related with age. Then, we correlated the muscle markers between themselves and separated the output into cardiac markers, non-specific muscle markers (proteins that belongs to skeletal muscle as well as other tissues) and specific muscle markers (proteins solely present in the skeletal muscle). The results are summarized in Table 5. Within the cardiac markers, there were moderate correlations between myoglobin and CK-MB as well as CK; cTnI was positively related with CK-MB and CK was moderately positively related with the CK-MB isoenzyme. Within the non-specific markers only myoglobin was positively correlated with all the other measured parameters (Table 5) and CK was positively related with AST. Interestingly, within the specific markers subset ssTnI did not correlate with any of the measured parameters including fsTnI, whereas fsTnI was weakly correlated with aldolase and AST, moderately/strongly correlated with myoglobin and strongly correlated with CPK (Table 5).

**Table 4:** Correlation between the muscle functionality/damage markers and clinical characteristics of patients in the whole population

**Variables**	**Age, r (P)**	**BMI, r (P)**
**ssTnI**	- 0.072 (0.622)	0.165 (0.263)
**fsTnI**	0.134 (0.352)	- 0.131 (0.371)
**Myoglobin**	0.292 (0.058)	- 0.026 (0.873)
**cTnI**	0.383 (0.012)	- 0.143 (0.372)
**CK-MB**	0.001 (0.994)	- 0.104 (0.513)
**CK**	0.078 (0.608)	0.007 (0.962)
**Aldolase**	0.030 (0.873)	- 0.122 (0.404)
**AST**	0.188 (0.192)	0.065 (0.659)
CK - creatine kinase. AST - aspartate aminotransferase. ssTnI - slow skeletal troponin I. fsTnI - fast skeletal troponin I. cTnI - cardiac troponin I. CK-MB - Creatine kinase-MB isoform. r – coefficient of correlation. P < 0.05 was considered statistically significant.		

**Table 5:** Correlation between the muscle functionality/damage markers divided into cardiac, non-specific and specific markers measured in the whole population

**Cardiac markers**	**Myoglobin, r (P)**	**cTnI, r (P)**	**CK-MB, r (P)**	**CK, r (P)**
Myoglobin	-			
cTnI	0.112 (0.479)	-		
CK-MB	0.346 (0.023)	0.486 (0.001)	-	
CK	0.542 (0.001)	0.016 (0.918)	0.391 (0.010)	-
**Non-specific markers**				
Myoglobin	-			
CK	0.542 (0.001)	-		
Aldolase	0.435 (0.004)	0.217 (0.147)	-	
AST	0.341 (0.025)	0.475 (0.001)	0.217 (0.127)	-
**Specific markers**				
ssTnI	- 0.012 (0.942)	- 0.138 (0.366)	- 0.038 (0.795)	0.252 (0.078)
fsTnI	0.639 (0.001)	0.711 (0.001)	0.342 (0.015)	0.375 (0.007)
CK - creatine kinase. AST - aspartate aminotransferase. ssTnI - slow skeletal troponin I. fsTnI - fast skeletal troponin I. cTnI - cardiac troponin I. CK-MB - Creatine kinase-MB isoform. r – coefficient of correlation. P < 0.05 was considered statistically significant. Specific markers identify those proteins solely present in the skeletal muscle whereas non-specific markers proteins that may be present also in other tissues.				

## Discussion

Our results confirmed that statin treatment was correlated with an increased average serum concentration of CK, whereas the other non-specific markers of muscles remained unchanged, suggesting the onset of a subclinical low-level muscular injury. This is in agreement with results from other randomized clinical trials where high dose statins produced an increase in circulating CK also in healthy subjects without any muscle-related complaints (*4*, *6*).

Nonetheless, our most important finding was that for the first time in humans we found an increase in fsTnI isoform, specific for fast-twitch fibers, in serum of subjects taking statins while the concentration of ssTnI was unchanged. Indeed, up to now only animal studies observed a fibre-specific effect of statins on muscle, with fast-twitch fibres more prone to ultrastructural and functional alterations induced by the drugs (*22*). Thus, the observation of different serum amounts of fiber-specific muscle damage markers between subjects using or not using statins may be considered as a clue for a differential release of the proteins from the muscle, reflecting the probable different susceptibility of fibers to the drugs. This is of paramount importance considering that other drugs, such as fibrates, seems to mainly target slow-twitch fibers (*23*, *24*). Therefore, the key point of our work was the detection of fiber-specific muscle damage through a simple blood test rather than muscle biopsy.

Nonetheless, although we observed a concomitant increase in both CK and fsTnI, the latter could be more sensitive in identifying low grade muscle injury than CK since a higher proportion of statin users were characterized by sub-clinically abnormal values of fsTnI (cut-off: > 75% than the median value). However, the paucity of human studies exploring the use of skeletal troponins, and in particular fsTnI, as muscle damage markers together with the lack of standardized analysis techniques precluded us to compare our data with normal values determined in a larger population. Therefore, the results we found might be a picture of our population and not generalizable to a larger one. Interestingly, the finding of a strong positive correlation between CK and fsTnI, observed also in another study not related with statins, suggests that the increase in CK could reflect the major susceptibility of fast-twitch fibres to statins as well (*17*). It has been reported that CK (subunit M) expression is increased in muscles composed mainly of fast-twitch fibres compared to those with slow-twitch, reflecting the higher anaerobic metabolism of Type II fibres (*25*). Therefore, it is not surprising that CK and fsTnI correlated, suggesting that both proteins more likely mark fast fibres. However, at this time it is still unknown what lies behind the increased susceptibility of fast-twitch fibres towards statins, although we can infer that differences in energetic metabolism as well as in structures involved in calcium release/reuptake can play major roles. Indeed, in a study Draeger and collaborators observed a breakdown of the T-tubular system, important for the transmission of action potential, in patients using statins (*26*). Considering that the fast-twitch fibres have a more extended and developed T-tubular system than slow-twitch fibres of the same species, it is tempting to speculate that this difference might also be reflected in the increased susceptibility of fast-twitch fibres, which in turn can be reflected in an increased leakage of fsTnI into the circulation (*27*). The lack of correlation or the presence of a weak correlation we found between several proteins examined may be due to the nature of the protein itself, thus non-specific or specific to skeletal muscle that may reflect the functionality of other organs and tissues (*e.g.* liver and cardiac muscle). In addition, the lack of correlation between the ssTnI and fsTnI subunits is not surprising since they are supposed to mark different skeletal fibres and therefore a correlation is not necessarily expected. Nonetheless, these lacking correlations do not undermine our hypothesis or our study conclusions.

This study was not without limitations. First, the already accounted small sample size may have weakened the generalization of our results to larger populations. However, we have to acknowledge that this is the first study evaluating both ssTnI and fsTnI separately and as specific markers of sub-clinical damage to skeletal muscles; therefore, it may be an important starting point for future studies. Second, the observational and cross-sectional design of the study precluded us to determine any cause-and-effect relationships between the measured variables as well as the extent of associations between skeletal troponin I isoforms and statin treatment. A longitudinal approach with an interventional design would be more valuable. Third, the population enrolled in this study may not reflect the real-life clinical application of the analysed protein markers, since relatively healthier subjects (*e.g.* dyslipidemic patients and those affected by metabolic syndrome) may be more appropriate for studying the statin-related muscle damage. However, our results may be a good starting point for a general applicability of the skeletal troponins for the evaluation of clinical/sub-clinical muscle damage regardless of the population. In addition, studies dealing with muscular markers and the evaluation of muscle functionality should acknowledge that statins may act as confounding factors.

In conclusion, our results suggest that fsTnI could be a good marker for monitoring statin-associated muscular damage outperforming traditional markers such as CK, opening new avenues for the evaluation of fibre-specific skeletal muscle damage. Further studies in larger cohorts are needed to confirm and extend the actual usefulness of both skeletal troponin I isoforms as markers for skeletal muscle damage associated with statin therapy.

## Supplementary material

Supplementary tables
