# Supplementary material for: Fast skeletal troponin I, but not the slow isoform, is increased in patients under statin therapy: a pilot study
Source: Biochem Med (Zagreb). 2018 Dec 15;29(1):010703. doi: 10.11613/BM.2019.010703 (PMC6294157; doi:10.11613/BM.2019.010703)
Supplement: Supplementary file 1 — Supplementary tables [file bm-29-1-010703-S1.pdf]

Supplementary table 1. Determination of cross-reactivity between ssTnI and fsTnI proteins assayed in the ELISA kits

	**+ssTnI 1500 pg/mL**	**+fsTnI 200 pg/mL**	**Blank**	**P**
**ELISA ssTnI**	1.209 ± 0.058	0.089 ± 0.003	0.092 ± 0.003	0.402[a]
**ELISA fsTnI**	0.068 ± 0.004	0.544 ± 0.044	0.066 ± 0.003	0.526[b]

Value represents the raw data (mean ± standard deviation) of a recombinant human ssTnI (included in the kit) or fsTnI (included in the kit) assayed in the fsTnI or ssTnI ELISA kit, respectively. The values did not differ with the blank, suggesting the lack of cross-reactivity. [a]Comparison +fsTnI *vs*. Blank. [b]Comparison +ssTnI *vs*. Blank.

Supplementary table 2. Raw values of the biochemical parameters determined in the study population (N = 51)

	Statin use		
	**NO (N = 37)**	**YES (N = 14)**	**P**
**CK (U/L)**	87.0 (40.0 - 203.5)	209.0 (62.5 - 863.0)	0.049
**Aldolase (U/L)**	4.3 (3.1 - 5.7)	5.5 (4.1 - 6.3)	0.104
**AST (U/L)**	1.7 (0.1 - 5.5)	3.6 (2.6 - 8.2)	0.106
**ssTnI (pg/mL)**	112.5 (26.5 - 262.4)	132.7 (41.9 - 1127.2)	0.370
**fsTnI (pg/mL)**	42.7 (9.6 - 116.9)	185.0 (55.7 - 526.9)	0.005
**Myoglobin (ng/mL)**	89.0 (40.0 - 211.0)	214.5 (82.0 - 2110.0)	0.037
**cTnI (ng/mL)**	0.042 (0.033 - 0.126)	0.124 (0.045 - 0.551)	0.045
**CK-MB (ng/mL)**	3.2 (2.2 - 7.2)	7.6 (3.5 - 25.1)	0.081

Data represent the raw median (interquartile range) of the biochemical parameters measured in the two groups not corrected for confounding factors. Comparisons were made by Mann-Whitney U test. IQR - interquartile range. CK - creatine phosphokinase. AST - aspartic aminotransferase. ssTnI - slow skeletal troponin I. fsTnI - fast skeletal troponin I. cTnI - cardiac troponin I. CK-MB - Creatine kinase-MB isoform. $P < 0.05$ was considered statistically significant.
